# Ionic Conductivity and Assembled Structures of Imidazolium Salt-Based Block Copolymers with Thermoresponsive Segments

**DOI:** 10.3390/polym9110616

**Published:** 2017-11-15

**Authors:** Kazuhiro Nakabayashi, Yu Sato, Yuta Isawa, Chen-Tsyr Lo, Hideharu Mori

**Affiliations:** 1 Department of Polymer Science and Engineering, Graduate School of Science and Engineering, Yamagata University, 4-3-16, Jonan, Yonezawa 992-8510, Japan; nakabayashi.k@yz.yamagata-u.ac.jp (K.N.); txa16692@st.yamagata-u.ac.jp (Y.S.); 2 Department of Organic Materials Science, Graduate School of Organic Materials Science, Yamagata University, 4-3-16, Jonan, Yonezawa 992-8510, Japan; tkh21629@st.yamagata-u.ac.jp (Y.I.); ct.lo@yz.yamagata-u.ac.jp (C.-T.L.)

**Keywords:** polymeric ionic liquid, ion conductivity, RAFT polymerization, block copolymer, thermoresponsive polymer

## Abstract

Ionic liquid-based block copolymers composed of ionic (solubility tunable)–nonionic (water-soluble and thermoresponsive) segments were synthesized to explore the relationship between ionic conductivity and assembled structures. Three block copolymers, comprising poly(*N*-vinylimidazolium bromide) (poly(NVI-Br)) as a hydrophilic poly(ionic liquid) segment and thermoresponsive poly(*N*-isopropylacrylamide) (poly(NIPAM)), having different compositions, were initially prepared by RAFT polymerization. The anion-exchange reaction of the poly(NVI-Br) in the block copolymers with lithium bis(trifluoromethanesulfonyl)imide (LiNTf_2_) proceeded selectively to afford amphiphilic block copolymers composed of hydrophobic poly(NVI-NTf_2_) and hydrophilic poly(NIPAM). Resulting poly(NVI-NTf_2_)-*b*-poly(NIPAM) exhibited ionic conductivities greater than 10^−3^ S/cm at 90 °C and 10^−4^ S/cm at 25 °C, which can be tuned by the comonomer composition and addition of a molten salt. Temperature-dependent ionic conductivity and assembled structures of these block copolymers were investigated, in terms of the comonomer composition, nature of counter anion and sample preparation procedure.

## 1. Introduction

Polymeric ionic liquids, which combine the advantages of macromolecules with various attractive features of ionic liquids, have been the subject of significant and sustained research [1,2,3,4,5]. Owing to a major concern of lithium-based batteries, recently, polymeric ionic liquids have been attractive as promising candidates to realize intrinsically safe rechargeable batteries. Ideally, polymeric ionic liquids can provide highly conductive and electrochemically stable membranes with a large amount of conducting lithium salts by an easy and industrially applicable process. One drawback of polymeric ionic liquids is insufficient ionic conductivity, which is generally several orders of magnitude less than pristine ionic liquid-type monomers. In order to overcome this important problem, many methodologies have been explored to design novel polymeric ionic liquids with complex architectures. An attractive approach is to use self-assembly of block copolymers with ionic liquid-based segments, by which the increased ionic conductivity can be occasionally obtained by effective confinement blocks within the ordered nanodomains [6,7,8,9,10,11]. The introduction of an ionic compound, either a solid salt (e.g., lithium salt) or a molten salt (e.g., ionic liquid), is another promising strategy to improve ion conductivity of polymeric ionic liquids [11,12,13].

Among the polymeric ionic liquids with complex architectures, imidazolium salt-based block copolymers have been widely studied, owing to their characteristic ionic conductivity and the ability to self-assemble into hierarchical structures [14,15,16]. A diverse range of ionic liquid-based block copolymers having various substituent groups and counterions has been developed using various controlled radical polymerizations, which involve atom-transfer radical polymerization [17,18], reversible addition–fragmentation chain transfer (RAFT) polymerization [19,20,21,22,23] and cobalt-mediated radical polymerization [24,25,26] of imidazolium-containing monomers. This has resulted in an understanding of the structure–morphology property correlation, including the ionic conductivity and nanostructured morphologies of self-assembled architectures [6,7,8,9]. Most of the systems based on the block copolymers consisted of a hydrophobic segment, such as styrene and methacryate, and a poly(ionic liquid) segment. In these systems, ion conduction is confined to the poly(ionic liquid) phase, whereas the hydrophobic phase imparts mechanical stability. In contrast, less attention has been paid to ionic liquid-based block copolymers containing hydrophilic segments, which may be due to the concern of deterioration of mechanical properties of the membrane with increasing water uptake.

Recently, new types of poly(ionic liquid)-containing block copolymers composed of hydrophilic segments have been explored, aiming to develop novel ion-conducting materials that can synergistically combine high ionic conductivity, high electrochemical stability and good mechanical properties [13,26,27]. Elabd et al. demonstrated the synthesis of a novel zwitterionic block copolymer with ionic conductivity (>1.5 mS cm^−1^ at 70 °C) [13], where one block contains a covalently attached cation and another block contains a covalently attached anion. Taton and Detrembleur et al. reported the synthesis of novel ion-conducting block copolymers with both blocks exclusively containing *N*-vinyl-imidazolium units, which exhibited ionic conductivity (1–3 × 10^−7^ S cm^−1^) and wide electrochemical stability [26]. In recent years, increasing attention has been also paid to thermoresponsive ionic gels as soft solids with high ionic conductivity and tunable mechanical strength, depending upon the temperature [28,29,30,31]. For example, thermoreversible polymer gels using a poly(ethylene oxide)-based triblock copolymer, which self-assembles into different microcrystalline phases by temperature change, have been developed as novel polymer gel electrolytes exhibiting microphase-dependent ionic conductivity [30]. Based on the unique combination of properties derived from ionic liquids and their temperature-sensitive nature, these thermoresponsive ionic gels can be considered as promising ion-conducting materials and polymeric electrolytes with tunable structures and ionic conductivity.

**Scheme 1:**
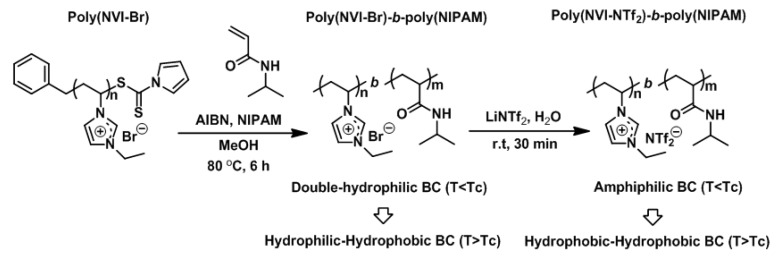
Synthesis of ionic liquid-based block copolymers (BCs) composed of poly(*N*-vinylimidazolium bromide) (poly(NVI-Br)) and poly(*N*-isopropylacrylamide) (poly(NIPAM)) by RAFT polymerization, followed by anion-exchange reaction. Tc (critical temperature) corresponds to the lower critical solution temperature, LCST, of the poly(NIPAM) segment (around 32 °C).

In this study, we describe the investigation of temperature-dependent ionic conductivity and assembled structures of ionic liquid-based block copolymers having a thermoresponsive segment, in terms of the comonomer composition, counteranion and sample preparation procedure. As shown in Scheme 1, poly(*N*-vinylimidazolium bromide) (poly(NVI-Br)) was chosen as an imidazolium salt-based poly(ionic liquid) segment, which can act as a solubility tuning component by changing the counteranion. As a hydrophilic (thermoresponsive) segment, we selected poly(*N*-isopropylacrylamide) (poly(NIPAM)), which exhibits a soluble–insoluble transition (lower critical solution temperature, LCST) at 32 °C in water [32,33]. As for block copolymers with ionic liquid and thermoresponsive blocks, there is capability to generate ideal morphology for ionic conduction to use their thermoresponsive nature efficiently. Double-hydrophilic block copolymers, poly(NVI-Br)-*b*-poly(NIPAM)s, having different comonomer compositions, were initially prepared by RAFT polymerization of NVI-Br using the dithiocarbamate-terminated poly(NIPAM) as a macro-chain transfer agent (CTA). The properties and solubility of the poly(NVI-Br) segment in the block copolymers can be tuned by changing the structure of the counteranion (hydrophilic Br^−^ ion and hydrophobic bis(trifluoromethanesulfonyl)imide ((SO_2_CF_3_)_2_N^−^ = Tf_2_N^−^) ion, respectively) via anion-exchange reactions. Hence, these block copolymers are regarded as ionic (solubility tunable)–nonionic (water-soluble and thermoresponsive) block copolymers that can be transformed from double hydrophilic block copolymers into amphiphilic ones via anion exchange. Since the poly(NIPAM) segment changes from a fully hydrated, expanded and soluble chain to a dehydrated, collapsed and insoluble chain in water at the critical temperature (Tc, which corresponds to the LCST of poly(NIPAM) chain), the double hydrophilic and amphiphilic block copolymers at room temperature become amphiphilic and double hydrophobic block copolymers above Tc (Scheme 1). The morphological control by using their thermoresponsive nature is expected in the films prepared from thermoresponsive block copolymers under different conditions. Such temperature-responsive properties should affect the ionic conductivity and assembled structures of the ionic liquid-based block copolymers developed in this study. The effect of the additional ionic liquid, alkylimidazolium salt (*N*-methyl ethylimidazolium bis(trifluoromethanesulfonyl)imide = MEI-NTf_2_), on the ion conductivity, was also investigated. We believe that this synthetic approach and the resulting ionic liquid-based block copolymers having the thermoresponsive segment will significantly extend the design of ionic conductive polymeric materials with predetermined hydrophilic/hydrophobic combinations, compositions and temperature-dependent assembled structures.

## 2. Experimental Section

### 2.1. Materials

*N*-Isopropylacrylamide (NIPAM, Tokyo Chemical Industry, Tokyo, Japan, 98%) was purified two times by recrystallization from *n*-hexane. 1-Ethyl-3-vinylimidazolium bromide (*N*-vinyl ethylimidazolium bromide, NVI-Br) was synthesized by the reaction of 1-vinylimidazole with bromoethane according to a previously reported method with slight modifications [19,34]. *N*-Methyl ethylimidazolium bis(trifluoromethanesulfonyl)imide (MEI-NTf_2_) was prepared by 1-methylimidazole with bromoethane, followed by the anion-exchange reaction (see Figures S1 and S2 for ^1^H and ^13^C NMR spectra, Supplementary Information). Lithium bis(trifluoromethanesulfonyl)imide (LiNTf_2_, Tokyo Chemical Industry, Tokyo, Japan, >98%) and other materials were used as received.

### 2.2. Synthesis of Block Copolymers

The block copolymers comprising poly(NVI-Br) as an ionic liquid-based segment and poly(NIPAM) as a hydrophilic segment were synthesized by RAFT polymerization of NIPAM using the dithiocarbamate-terminated poly(NVI-Br) macro-CTAs, which were prepared by RAFT polymerization using benzyl 1-pyrrolecarbodithioate. A detailed description of the synthesis procedure can be found in an earlier publication [19]. The polymerization of NIPAM was conducted in the presence of the dithiocarbamate-terminated poly(NVI-Br) at different monomer-to-CTA ratios ([NIPAM]_0_/[macro-CTA]_0_ = 50–400) in methanol at 80 °C for 6 h (Table S1, Supplementary Information). The copolymer composition was determined using ^1^H NMR spectroscopy by a comparison of peaks associated with the two comonomers.

### 2.3. Anion-Exchange Reactions of the Block Copolymers

The anion exchange to replace Br^−^ with NTf_2_^−^ in the block copolymer was carried out according to the method used for the exchange reaction of poly(*N*-vinylimidazolium bromide), with slight modifications [34]. A representative example (NIPAM/NVI-Br = 52/48 mol %) is as follows: To a solution of poly(NVI-Br)-*b*-poly(NIPAM) (0.1 g, 0.48 monomer unit mmol) dissolved in distilled water (1.0 mL), an aqueous solution (1.0 mL) of lithium bis(trifluoromethanesulfonyl)imide (LiNTf_2_, 0.6 g, 2.1 mmol) was added dropwise. The mixture was then stirred at room temperature for 30 min. The precipitate was isolated by filtration, and the residual solid was washed with distilled water. Subsequently, the product was dried under vacuum at 60 °C overnight to afford poly(NVI-NTf_2_)-*b*-poly(NIPAM) as a white solid (0.121 g, 88%).

^1^H NMR (400 MHz, DMSO-*d*_6_, ppm): δ 0.9–1.1 (broad, 3H, CH–(C*H*_3_)_2_ in the side chain of NIPAM), 1.2–1.5 (broad, 3H, –CH_2_–C*H*_3_ in the side chain of NVI-NTf_2_), 1.4–2.6 (broad, 2H, –CH–C*H*_2_– in the main chain of NVI-NTf_2_ and broad, 3H, –C*H*–C*H*_2_– in the main chain of NIPAM), 3.6–3.8 (broad, 1H, –C*H*–(CH_3_)_2_ in the side chain of NIPAM), 3.8–4.2 (broad, 2H, –C*H*_2_–CH_3_ in the side chain of NVI-NTf_2_), 4.2–4.5 (broad, 1H, –C*H*–CH_2_– in the main chain of NVI-NTf_2_), 6.8–8.1 (broad, 1H, –C*H*=C*H*– in the side chain of NVI-NTf_2_ and –N*H*– in the side chain of NIPAM), 8.4–9.2 (broad, 1H, N–C*H*–N in the side chain of NVI-NTf_2_) ppm.

^13^C NMR (400 MHz, DMSO-*d*_6_, ppm): δ 15.1 (–CH_2_–*C*H_3_ in the side chain of NVI-NTf_2_), 22.8 (–CH–(*C*H_3_)_2_ in the side chain of NIPAM), 33–38 (–*C*H_2_–CH– in the main chain of NVI-NTf_2_ and –*C*H_2_–*C*H– in the main chain of NIPAM), 45.3, 45.5 (–*C*H_2_–CH_3_ in NVI-NTf_2_ and –*C*H–(CH_3_)_2_ in NIPAM), 52-57 (–CH_2_–*C*H– in the main chain of NVI-NTf_2_), 115.2, 118.4, 121.6, 124.8 (–SO_2_–*C*F_3_), 120.9 (CH=*C*H–N), 123.9 (–N–*C*H=CH), 135.3 (N–*C*H–N), 173.8 (O=*C*–NH) ppm.

^1^H and ^13^C NMR spectra, and the solubility of these block copolymers, are shown in Figures S3 and S5, and Table S2, respectively (see Supplementary Information).

### 2.4. Instrumentation

^1^H (400 MHz) and ^13^C NMR (100 MHz) spectra were recorded with a JEOL JNM-ECX400 (JEOL, Akishima, Japan). Elemental analysis was carried out on a Perkin-Elmer 2400 II CHNS/O analyzer (Perkin-Elmer, Waltham, MA, USA). For the determination of the number-average molecular weight (*M*_n_) and molecular weight distribution (*M*_w_/*M*_n_) of the poly(*N*-vinylimidazolium bromide)-based block copolymers, the SEC measurement was conducted using special SEC columns (Tosoh, Tokyo, Japan) applicable for cationic polymers in acetonitrile/water (50/50 vol %) containing 0.05 M NaNO_3_ as an eluent. The SEC was performed on a system consisting of a Tosoh DP-8020 pump (Tosoh, Tokyo, Japan) and a Viscotek TDA model-301 triple detector array (RI, Viscosity, and RALLS; wavelength = 670 nm) (Malvern, Worcestershire, UK) at a flow rate of 1.0 mL/min. The column setup was as follows: two consecutive columns (Tosoh TSK-GELs (exclusion limited molecular weight): G5000PW_XL_-CP (1 × 10^6^), G3000PW_XL_-CP (9 × 10^4^), 30 cm each) and a guard column (TSK-guardcolumn PW_XL_-CP, 4.0 cm) (Tosoh, Tokyo, Japan). Poly(ethylene oxide) standards, which are commonly used as standards in the SEC system with a H_2_O-based eluent, were employed for calibration. Thermal gravimetric (TG) analysis was performed on a SEIKO SSC6200 (Hitachi High-Technologies, Tokyo, Japan) at a heating rate of 10 °C /min under a nitrogen atmosphere. Tapping mode scanning force microscopy (SFM) observation was performed with an Agilent AFM 5500 (Agilent Technologies, Santa Clara, CA, USA), using micro-fabricated cantilevers with a force constant of approximately 34 N/m. The samples were prepared by the drop casting of polymer solutions onto mica substrates.

Ionic conductivity in the planar direction of a membrane was determined using an electrochemical impedance spectroscopy technique over the frequency from 5 to 10^5^ Hz (Hioki 3532-80, Hioki, Ueda, Japan) at 25, 55 and 90 °C under the ambient humidity conditions. A two-point-probe conductivity cell with two platinum plate electrodes was fabricated. The cell was placed under a thermo-controlled chamber. Ionic conductivity (σ) was calculated from:

σ = *d*/(*L*_s_*w*_s_*R*),

where *d* is the distance between the two electrodes, *L*_s_ and *w*_s_ are the thickness and width of the membrane, and *R* is the resistance value measured.

## 3. Results and Discussion

### 3.1. Synthesis and Anion-Exchange Reaction of Imidazolium Salt-Based Block Copolymers

**Figure 1:**
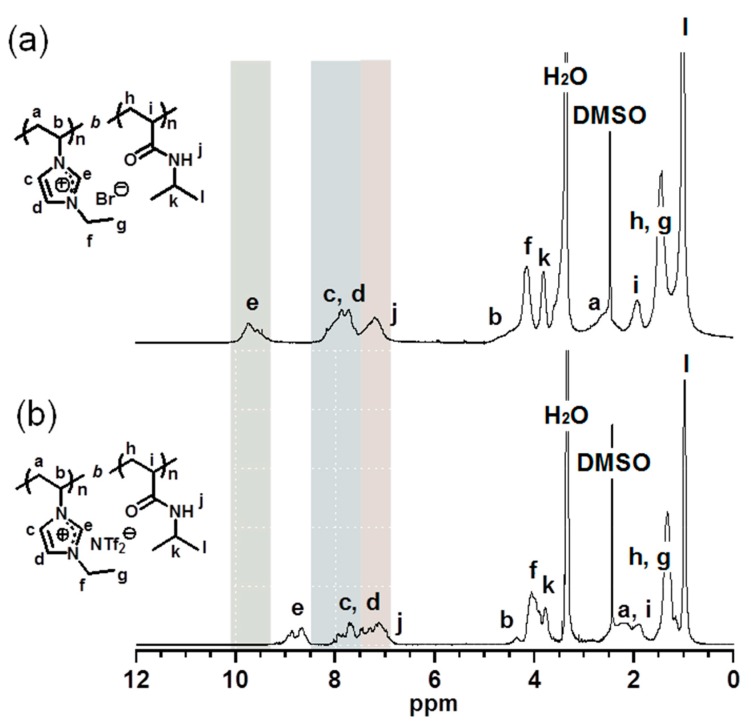
^1^H NMR spectra (DMSO-*d*_6_) of (**a**) poly(NVI-Br)-*b*-poly(NIPAM) and (**b**) poly(NVI-NTf_2_)-*b*-poly(NIPAM).

Two types of imidazolium salt-based block copolymers having ionic (solubility tunable) and nonionic (thermoresponsive) segments were employed as double-hydrophilic and amphiphilic block copolymers (Scheme 1). We focused on the design and manipulation of assembled structures of the imidazolium salt-based block copolymers and their ion conductivity. Previously, we reported the synthesis of imidazolium-based block copolymers involving a hydrophilic poly(NIPAM) segment by RAFT polymerization of NIPAM from the poly(NVI-Br) macro-CTA [19]. In this study, the same synthetic approach was employed for the preparation of the block copolymers composed of hydrophilic poly(NVI-Br) and thermoresponsive poly(NIPAM). In order to obtain the block copolymers having pre-determined comonomer compositions and molecular weights, the monomer-to-CTA ratio was varied in the range of [NVI-Br]_0_/[macro-CTA]_0_ = 50–400. Under the given conditions, the NVI-Br content in the block copolymer was adjusted to be 21–68%, which was confirmed by ^1^H NMR spectroscopy (Figure 1a). In all cases, block copolymers with relatively low polydispersities were obtained, as shown in Table 1. The SEC traces of the block copolymer and poly(NVI-Br) macro-CTA also supported the successful synthesis of the desired block copolymer (Figure S4, Supplementary Information). The resulting block copolymer was soluble in H_2_O, DMSO and methanol, but insoluble in common organic solvents, such as acetone, THF, chloroform and ethyl acetate (Table S2, Supplementary Information), suggesting that the resulting product corresponds to a double-hydrophilic block copolymer. 

Anion exchange to replace Br^−^ with NTf_2_^−^ in the block copolymer was carried out according to the method used for the synthesis of poly(*N*-vinylimidazolium salt)s with slight modifications (Scheme 1) [34]. Poly(NVI-NTf_2_)-*b*-poly(NIPAM) was synthesized by initially dissolving poly(NVI-Br)-*b*-poly(NIPAM) in distilled water, after which an aqueous solution of LiN(SO_2_CF_3_)_2_(LiNTf_2_) was added dropwise to the polymer solution at room temperature. After stirring the mixture at room temperature for 30 min, the precipitate, which was formed by the anion-exchange reaction, was isolated by filtration. The residual solid was washed with distilled water to afford poly(NVI-NTf_2_)-*b*-poly(NIPAM), which was soluble in acetone and DMSO, independent of the comonomer composition (Table S2, Supplementary Information). The structure of the anion-exchanged block copolymer was confirmed by ^1^H and ^13^C NMR measurements. The ^1^H NMR spectrum of poly(NVI-NTf_2_)-*b*-poly(NIPAM) exhibited peaks at 8.4–9.2 ppm attributed to the imidazolium ring, which were notably shifted from the peak at 9.0–10 ppm corresponding to the original poly(NVI-Br) unit, while the peaks attributed to the poly(NIPAM) segment were unchanged even after the exchange reaction (Figure 1). Similar peak shift of the imidazolium unit was also observed in the corresponding homopolymers (Figure S3, Supplementary Information). Successful incorporation of the NTf_2_ unit into poly(NVI-Br)-*b*-poly(NIPAM) was also confirmed by the presence of the four peaks at 115–125 ppm in ^13^C NMR spectrum of poly(NVI-NTf_2_)-*b*-poly(NIPAM), corresponding to the trifluoromethyl group (Figure S5, Supplementary Information). The exchanged block copolymers having the NTf_2_ counteranion were difficult to dissolve in water (Table S2, Supplementary Information), indicating successful transformation of the double-hydrophilic block copolymers into amphiphilic block copolymers by the anion-exchange reaction. In other words, these results indicate that anion exchange of the poly(NVI-Br) segment in the block copolymers proceeds selectively to afford amphiphilic block copolymers composed of a hydrophobic poly(NVI-NTf_2_) and a hydrophilic nonionic segment, poly(NIPAM). The thermal stability of the block copolymers was evaluated by TG analysis (Figure S6, Supplementary Information). The temperature to start the decomposition depended on the anion; both block copolymers with the Br and NTf_2_ anions exhibited high thermal stability (i.e., the 5 wt % decomposition temperature over 250 °C).

### 3.2. Self-Assembly and Ionic Conductivity of Amphiphilic Block Copolymers

**Figure 2:**
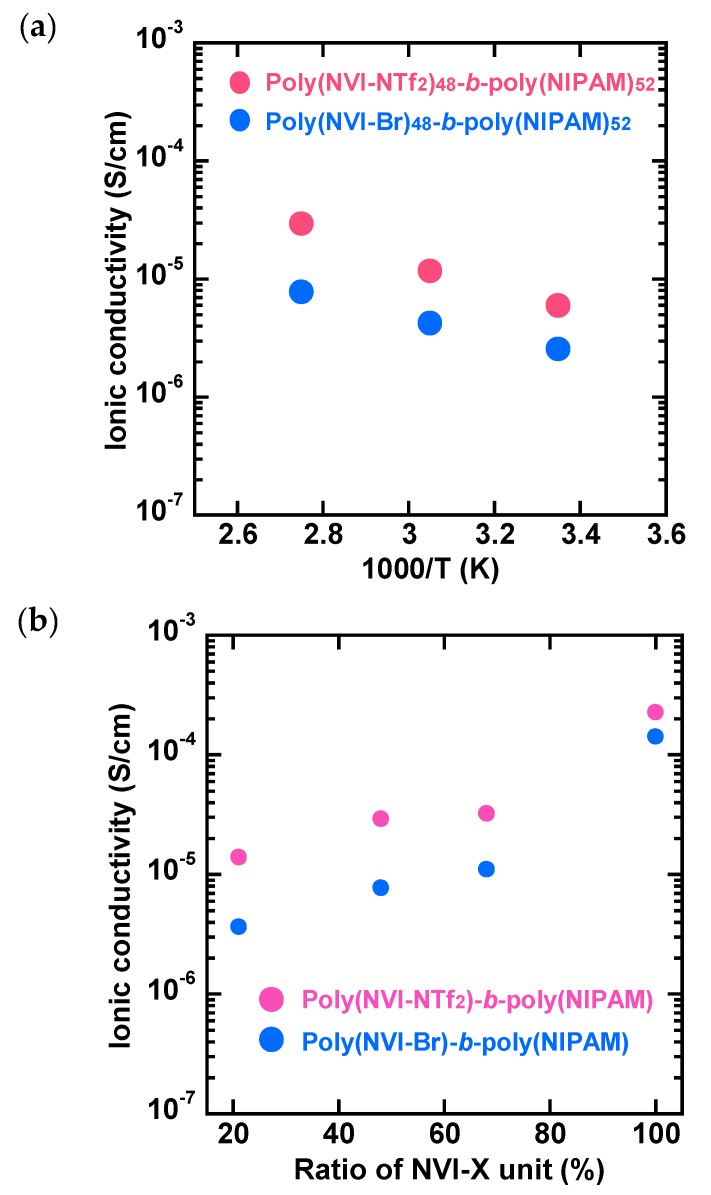
(**a**) Temperature-dependent ionic conductivity of poly(NVI-Br)_48_-*b*-poly(NIPAM)_52_ and poly(NVI-NTf_2_)_48_-*b*-poly(NIPAM)_52_, and (**b**) composition-dependent ionic conductivity of poly(NVI-NTf_2_)-*b*-poly(NIPAM)s and poly(NVI-Br)-*b*-poly(NIPAM)s at 90 °C. Acetone solutions of poly(NVI-NTf_2_)-*b*-poly(NIPAM)s were cast onto a platinum electrode and dried at 40 °C for 2 h. Similarly, methanol solutions were employed for poly(NVI-Br)-*b*-poly(NIPAM)s.

The temperature-dependent ionic conductivities of the amphiphilic block copolymers, poly(NVI-NTf_2_)-*b*-poly(NIPAM)s, obtained after the exchange reaction, were studied under the ambient humidity conditions. Here, acetone was selected as a solvent for sample preparation, as it has good solubility for the amphiphilic block copolymers, in which both poly(NIPAM) and poly(NVI-NTf_2_) segments are dissolved independently of the comonomer composition. Acetone solutions of the amphiphilic block polymers were cast onto a platinum electrode and dried at 40 °C for 2 h. As shown in Figure 2a, poly(NVI-NTf_2_)_48_-*b*-poly(NIPAM)_52_ exhibits ionic conductivities of 2.9 × 10^−5^, 1.2 × 10^−5^ and 5.9 × 10^−6^ S/cm at 90, 55 and 25 °C, respectively, which are apparently 2–4-fold higher than those of pristine poly(NVI-Br)_48_-*b*-poly(NIPAM)_52_. As shown in Figure 2b, poly(NVI-NTf_2_)-*b*-poly(NIPAM)s exhibited ionic conductivities higher than those of poly(NVI-Br)-*b*-poly(NIPAM)s, regardless of the comonomer composition (NVI-X content). This is an indication that the ionic conductivity of poly(NVI-X)-*b*-poly(NIPAM) was strongly dependent on the counteranions, as in the case of various previously reported polymers containing ionic-liquid moieties [35,36,37,38,39].

**Figure 3:**
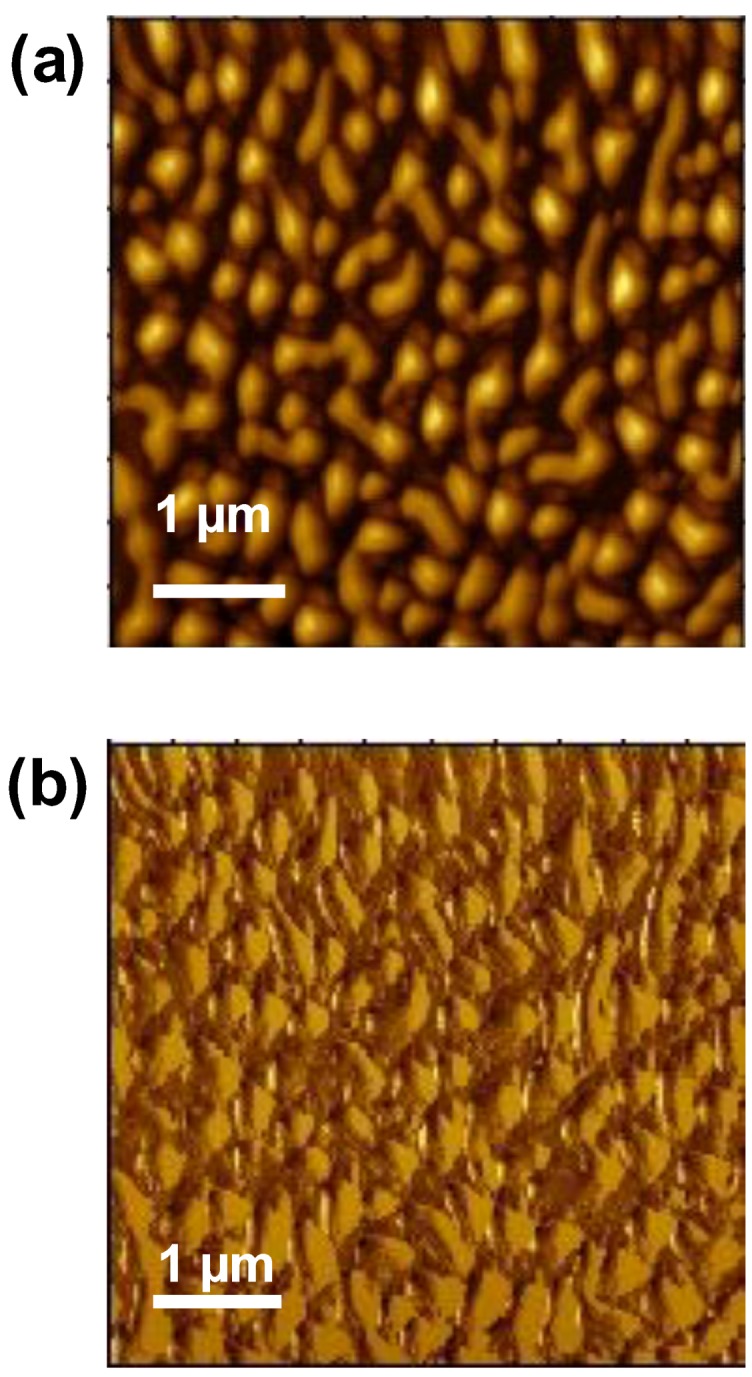
SFM (**a**) height (z-range = 40 nm) and (**b**) phase (z-range = 60°) images of poly(NVI-NTf_2_)_48_-*b*-poly(NIPAM)_52_. The sample was prepared by drop-casting of the acetone solution of the amphiphilic block copolymer onto mica substrates.

The tapping mode height and phase image of the surface of the poly(NVI-NTf_2_)_48_-*b*-poly(NIPAM)_52_, as a representative sample, was recorded under ambient conditions on the 5 × 5 μm^2^ scale to investigate the phase separation between the hydrophilic and hydrophobic domains (Figure 3). The bright and dark regions were assigned to the soft structure corresponding to the hydrophilic poly(NIPAM) domains and the hard structure corresponding to the hydrophobic poly(NVI-NTf_2_) domains, respectively. In the case of ion-conducting materials, the formation of continuous domains in the nano scale is crucial, because continuous domains can enhance the ion conductivity. As can be seen in Figure 3a, the dark poly(NVI-NTf_2_) domains were well dispersed and connected to each other. On the other hand, no detectable phase separation was observed on the surface of the double-hydrophilic block copolymer, poly(NVI-Br)_48_-*b*-poly(NIPAM)_52_. The size of each domain was several hundred nanometers (100–300 nm), and they were formed by the assembly of hydrophilic and hydrophobic blocks. The volume fraction of the poly(NVI-NTf_2_)_48_-*b*-poly(NIPAM)_52_ is considered to correspond to the lamellar phase. However, the exact evaluation of the volume fraction was difficult because of the presence of the bulky NTf_2_ anion in poly(NVI-NTf_2_), which should be different from the Br anion in poly(NVI-Br). In other words, the anion exchange to replace Br^−^ with NTf_2_^−^ in the block copolymer leads to a change in the hydrophilicity (solubility) and volume fraction of the poly(ionic liquid) segment in the block copolymers, as well as the Flory–Huggins interaction parameters between poly(NVI-Br) and poly(NVI-NTf_2_) against poly(NIPAM). Nevertheless, the hydrophilic–hydrophobic interaction strongly affected the morphology of poly(NVI-NTf_2_)-*b*-poly(NIPAM) films. The continuous poly(NVI-NTf_2_) domains of poly(NVI-NTf_2_)-*b*-poly(NIPAM) functioned as the ionic conductive path, which indeed contributed to the enhancement of the ionic conductivity. This fact demonstrated that block copolymers with a well-defined hydrophilic–hydrophobic structure are potentially promising polymer architectures for achieving high ionic conductivity.

**Figure 4:**
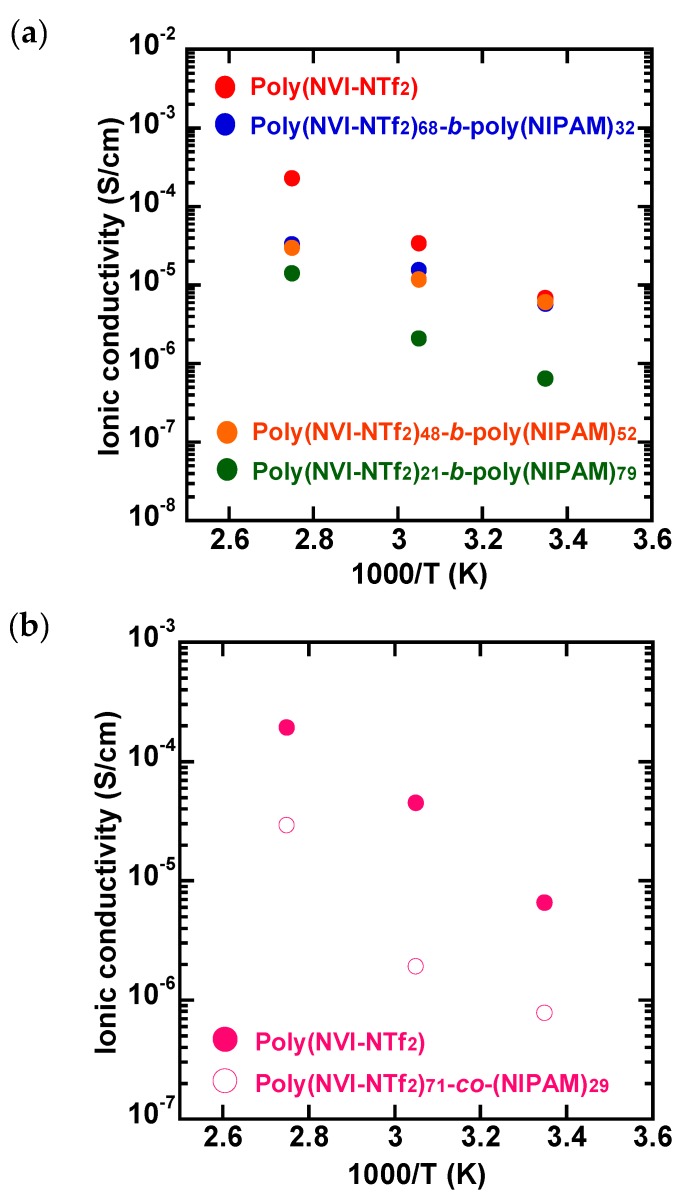
Temperature-dependent ionic conductivity of (**a**) poly(NVI-NTf_2_)-*b*-poly(NIPAM)s having different comonomer compositions and (**b**) poly(NVI-NTf_2_) and poly(NVI-NTf_2_-*co*-NIPAM).

Figure 4a shows the relationship between the NVI-NTf_2_/NIPAM ratio and the temperature-dependent ionic conductivity of poly(NVI-NTf_2_)-*b*-poly(NIPAM) films cast from the acetone solutions. As expected, the comonomer composition of the block copolymer has a significant influence on the ionic conductivity of poly(NVI-NTf_2_)-*b*-poly(NIPAM). For example, the block copolymer having higher NVI-NTf_2_ content (NVI-NTf_2_/NIPAM = 68/32) exhibited 2–4-fold higher ionic conductivity in the range of 90 to 25 °C, in comparison with the copolymer having lower NVI-NTf_2_ content (NVI-NTf_2_/NIPAM = 21/79). The observed result demonstrated that the poly(NVI-NTf_2_)/poly(NIPAM) block ratios clearly affected the ionic conducting behavior, which might result from the difference in the charge-carrier concentration and phase-separation behavior. As a control experiment, the ionic conductivity of poly(NVI-NTf_2_) prepared independently by RAFT polymerization of NVI-Br and a subsequent anion-exchange reaction was evaluated at three different temperatures under the same conditions. As shown in Figure 4a, the ionic conductivities of poly(NVI-NTf_2_)-*b*-poly(NIPAM)s having higher NVI-NTf_2_ content (NVI-NTf_2_/NIPAM = 68/32 and 48/52) were comparable to that of the control sample, poly(NVI-NTf_2_), at 25 °C. In the case of the homopolymer, the ionic conductivity increased about two orders of magnitude, when increasing the temperature from 25 to 90 °C. In contrast, the conductivity increase with elevated temperatures is less pronounced for poly(NVI-NTf_2_)-*b*-poly(NIPAM)s, which may be due to less temperature-dependent segmental motion of the block copolymer originating from the incorporation of the poly(NIPAM) segment.

A random copolymer, poly(NVI-NTf_2_-*co*-NIPAM), prepared by RAFT copolymerization of NVI-NTf_2_ and NIPAM, was also employed as another control sample. As can be seen in Figure 4b, poly(NVI-NTf_2_-*co*-NIPAM) exhibited lower ionic conductivity than the homopolymer, and no significant difference was detected in the conductivity with elevated temperatures. In comparison to the corresponding block copolymer, poly(NVI-NTf_2_)_68_-*b*-poly(NIPAM)_32_, a difference in temperature-dependent ionic conductivity was observed in the random copolymer. Poly(NVI-NTf_2_)_71_-*co*-(NIPAM)_29_ exhibited much lower ionic conductivity at 25 and 55 °C compared to poly(NVI-NTf_2_)_68_-*b*-poly(NIPAM)_32_, although the ionic conductive component (NVI-NTf_2_ content) was almost the same for both. As discussed earlier, the block copolymer exhibited clear phase separation in the nano scale due to the well-defined hydrophilic–hydrophobic block structure. Continuous poly(NVI-NTf_2_) blocks functioned as the ionic conductive path in the block copolymer, which contributed to the improvement of the ionic conductivity [40,41,42]. On the other hand, the random copolymer had no capacity to form phase separation in the nano scale. The difference in phase-separation capacity was clearly reflected in the ionic conductivity behavior.

In order to confirm the effect of the sample annealing, we attempted to evaluate the ion conductivity of the samples treated at higher temperatures (90 and 130 °C). As can be seen in Figure S7 (Supplementary Information), the thermal treatment of the sample at 90 °C for 2 h had no significant influence on the ionic conductivities. Further increase in the temperature (130 °C for 2 h) led to the detachment of the sample and the platinum plate electrodes from the glass substrate, resulting in the impossibility of measuring. Occasionally, it was hard to prepare smooth films dried at higher temperature (>100 °C). In order to obtain information on molecular motion, the glass transition temperature (*T*_g_) of the block copolymer, poly(NVI-NTf_2_)-*b*-poly(NIPAM), was evaluated, in addition to the corresponding homopolymer, poly(NVI-NTf_2_). As shown in Figure S8 (Supplementary information), the *T*_g_ value of poly(NVI-NTf_2_) was 63 °C (lit. [35] *T*_g_ = 56 °C), which was comparable to the block copolymer. The *T*_g_ values of poly(NIPAM)s were reported to be in the range of 115–145 °C, depending on the molecular weight and tacticity [43]. Therefore, the molecular motion of the block copolymer was restricted, even after annealing treatment at 90 °C. Further investigations aiming to manipulate the morphology of the block copolymers by tuning the comonomer composition, counteranion and the sample preparation conditions are now in progress and will be reported elsewhere. 

**Figure 5:**
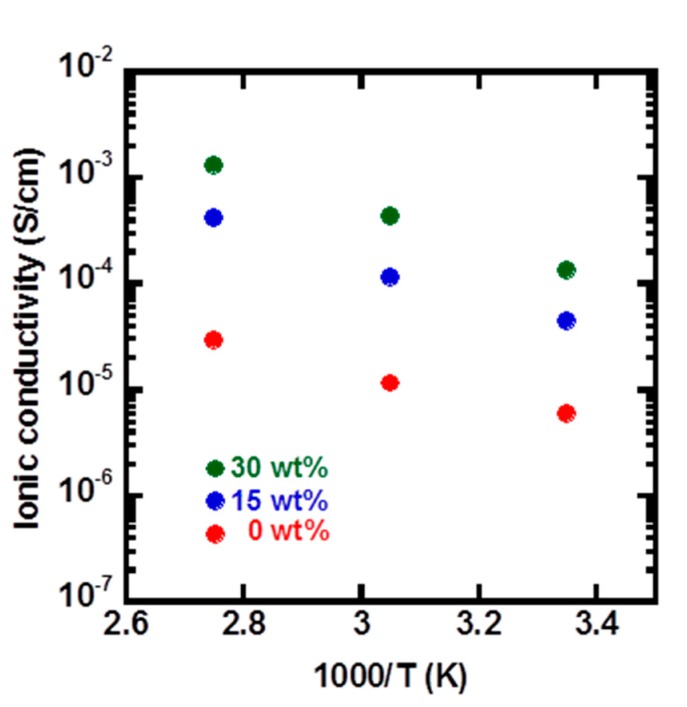
Temperature-dependent ionic conductivity of poly(NVI-NTf_2_)_48_-*b*-poly(NIPAM)_52_ containing different amounts of the ionic liquid (MEI-NTf_2_).

The introduction of a molten salt, such as an ionic liquid, has been extensively employed as an effective approach to improve ionic conductivity of polymeric ionic liquids [11,12,13]. By introducing the ionic liquid, an enhanced segmental motion of the polymer chain can be obtained, which leads to higher ionic conductivity. Here, the effect of *N*-methyl ethylimidazolium bis(trifluoromethanesulfonyl)imide (MEI-NTf_2_) on the ionic conductivity of poly(NVI-NTf_2_)-*b*-poly(NIPAM) was investigated in the range of 90 to 25 °C. The ionic conductivity of poly(NVI-NTf_2_)-*b*-poly(NIPAM) with MEI-NTf_2_ tended to be higher than that without the ionic liquid. As shown in Figure 5, in the presence of 30 wt % MEI-NTf_2_, high ionic conductivities of 1.34 × 10^−3^, 4.33 × 10^−4^ and 1.34 × 10^−4^ S/cm were achieved with poly(NVI-NTf_2_)_48_-*b*-poly(NIPAM)_52_ at 90, 55 and 25 °C, respectively. The values were more than 10-times higher than those of the original amphiphilic block copolymer without the ionic liquid, and significant increase in the ionic conductivity by the addition of MEI-NTf_2_ was detected, independent of the temperature. The ionic conductivity of polymers containing ionic liquid moieties ranged from ca. 10^−7^ to 10^−4^ S/cm below 90 °C in most reported examples [35,36,37,38,39]. The observed result demonstrated that the addition of the ionic liquid clearly affected the ionic conducting behavior, which might result from enhanced segmental molecular motion of the polymer chain. In the system, the presence of the molten salt may help to enhance the ionic conductive path due to the chemical affinity of the two components, poly(NIPAM) and poly(NVI-NTf_2_) blocks.

### 3.3. Self-Assembly and Ionic Conductivity of Double-Hydrophilic Block Copolymers

**Figure 6:**
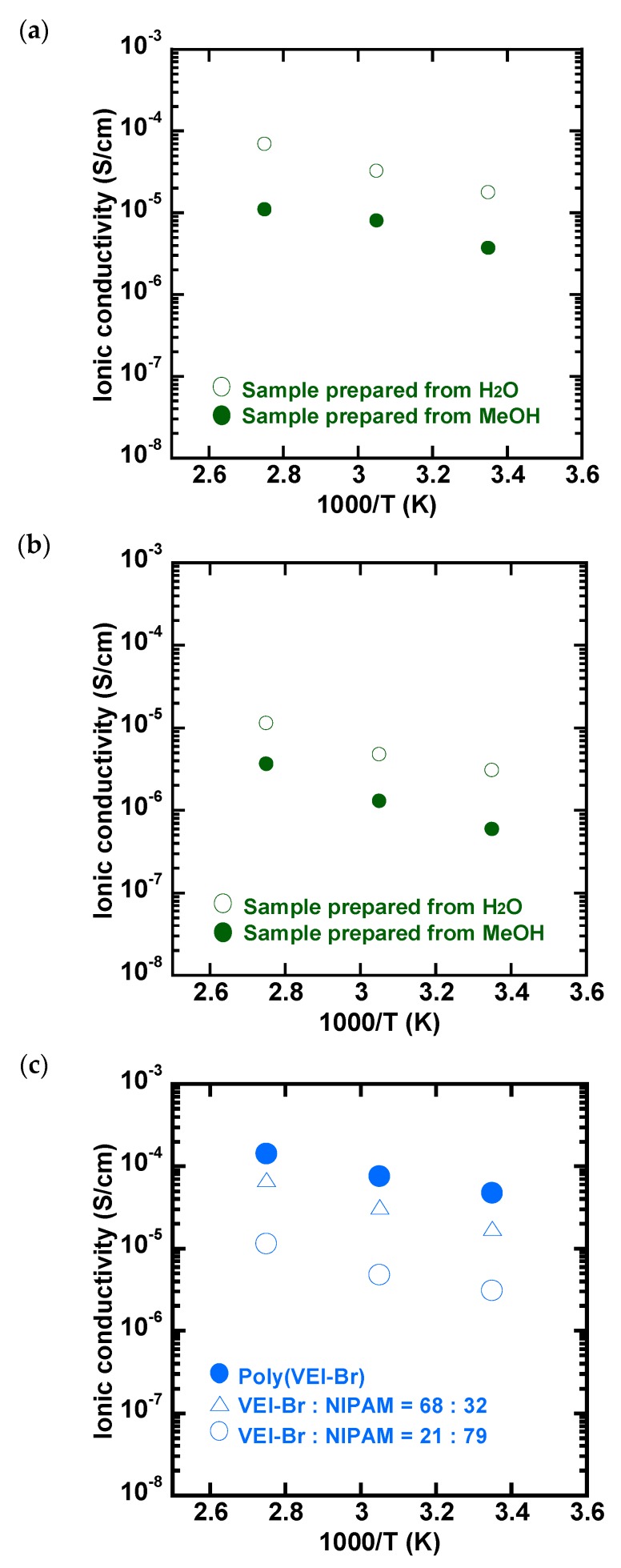
Preparation-dependent ionic conductivity of poly(NVI-Br)-*b*-poly(NIPAM)s; NVI-Br/NIPAM = (**a**) 68/32 and (**b**) 21/79. Hot aqueous and MeOH solutions of the block copolymers (40 °C) were cast onto a platinum electrode and dried at 40 °C for 2 h. (**c**) Temperature-dependent ionic conductivity of poly(NVI-Br)-*b*-poly(NIPAM)s having different comonomer compositions.

The temperature-dependent ionic conductivities of the double-hydrophilic block copolymers, poly(NVI-Br)-*b*-poly(NIPAM)s, were studied under the ambient humidity conditions. Initially, the effect of the sample preparation method on the ion conductivity was evaluated. An aqueous solution of the double-hydrophilic block copolymer, poly(NVI-Br)_48_-*b*-poly(NIPAM)_52_, was dissolved in water at 40 °C. Then, the hot aqueous solution was drop-cast onto a platinum electrode and dried at 40 °C. As a comparison, the sample was prepared from methanol solution at 40 °C. As shown in Figure 6, the samples cast from the heated aqueous solution exhibited 4–8-times higher ionic conductivity in the range of 90 to 25 °C. This may be due to the difference in assembled structures of the block copolymers in the thin films. Indeed, the micelles consisting of a relatively hydrophilic shell of poly(NVI-Br) and a hydrophobic core of the dehydrated poly(NIPAM) can be formed in water at 40 °C (above LCST). The dynamic light scattering (DLS) analysis of the poly(NVI-Br)_48_-*b*-poly(NIPAM)_52_ aqueous solution at 40 °C supported the micelle formation (Figure S9, Supplementary Information). The assembly of hydrophilic poly(NVI-Br) shells would function as a continuous ion conductive path.

**Figure 7:**
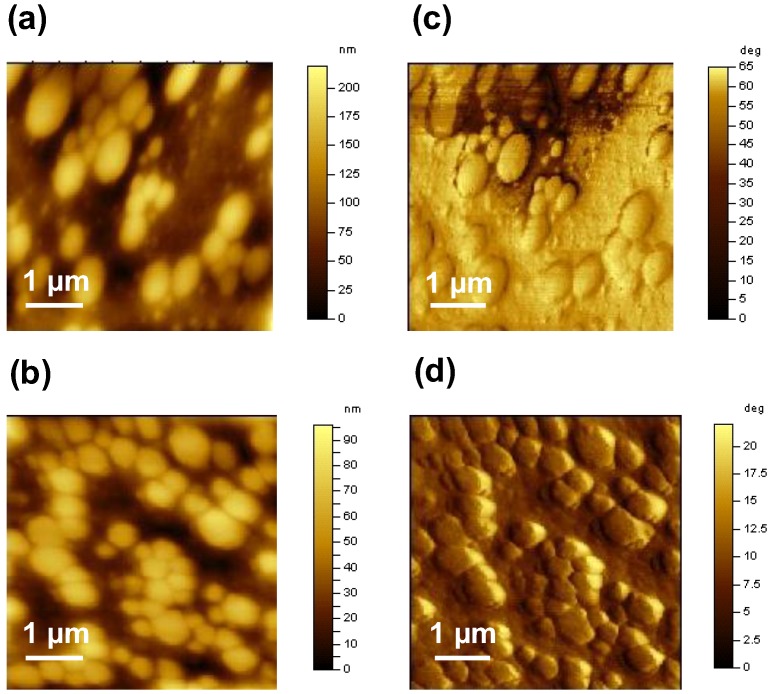
SFM (**a**,**b**) height and (**c**,**d**) phase images of poly(NVI-Br)-*b*-poly(NIPAM)s; NVI-Br/NIPAM = (**a**,**c**) 68/32 and (**b**,**d**) 48/52. The samples were prepared by the drop casting of the aqueous solutions of the block copolymers at 40 °C onto mica substrates.

As shown in Figure 7, assembled structures are visible in SFM images of the samples prepared from aqueous solutions of the double-hydrophilic block copolymer at 40 °C. The dark and bright parts in the height images may correspond to poly(NVI-Br) and poly(NIPAM) domains, respectively. On the other hand, such an assembled structure was not observed in the sample prepared from methanol solution (Figure S10, Supplementary Information). The lower confinement of ionic sites in the sample film might lead to the lower ionic conductivity of poly(NIPAM)-*b*-poly(NVI-Br) compared to the sample prepared from the hot aqueous solution. Morphological control under the proper conditions by using the LCST feature of the poly(NIPAM) block was an interesting method for the enhancement of ionic conductivity. We believe that this simple morphological control by the thermoresponsive nature of the block copolymer is an advantage of the combination of block copolymers with the thermoresponsive block. Furthermore, the ionic conductivity of poly(NVI-NTf_2_)-*b*-poly(NIPAM) can be also enhanced by utilizing this method.

## 4. Conclusions

The control of the polymer structure in block copolymers, such as the hydrophilic/hydrophobic feature and block lengths, is an essential issue for ionic conductive materials because they contribute to the formation of adjusted phase separation, which leads to the enhancement of ionic conductivities. To investigate the correlation between polymer structure and ionic conductivity, two block copolymers, poly(NVI-Br)-*b*-poly(NIPAM) and poly(NVI-NTf_2_)-*b*-poly(NIPAM), were initially prepared by RAFT polymerization and following an anion-exchange reaction. Poly(NVI-NTf_2_)-*b*-poly(NIPAM) exhibited 2–4-fold higher ionic conductivity in the range of 25–90 °C compared to poly(NVI-Br)-*b*-poly(NIPAM), which resulted from the continuous phase separation in the nano scale (ionic conductive path) between hydrophobic poly(NVI-NTf_2_) and hydrophilic poly(NIPAM) blocks. As for poly(NVI-Br)-*b*-poly(NIPAM) with the hydrophilic–hydrophilic blocks, the morphological control by using the LCST feature of poly(NIPAM) blocks resulted in an improvement of ionic conductivity. Consequently, these results demonstrated a clear correlation between the polymer structure in block copolymers, nano-morphology and ionic conductive behavior. By utilizing the suitable block copolymer architecture, the development of promising ionic conductive materials based on imidazolium salt components is expected.

## Figures and Tables

**Table 1:** Characterization data of double-hydrophilic and amphiphilic block copolymers (BCs) prepared by RAFT polymerization, followed by anion-exchange reaction ^a^.

Composition ^b^ (NVI/NIPAM)	*M*_w_/*M*_n_^c^	Double-Hydrophilic BC *M*_n_, ^d^ Poly(NVI-Br)/Poly(NIPAM)	Amphiphilic BC *M*_n_*,* ^d^ Poly(NVI-NTf_2_)*/*Poly(NIPAM)
68/32	1.39	13,100/6300	26,000/6300
48/52	1.26	6100/6600	12,100/6600
21/79	1.38	9800/36,900	19,500/36,900

^a^ See Scheme 1 and Table S1 (Supplementary Information) for detailed polymerization conditions. ^b^ Calculated by ^1^H NMR in DMSO-*d*_6_. ^c^ Molecular weight distributions of poly(NVI-Br)-*b*-poly(NIPAM)s were measured by SEC using poly(ethylene oxide) standards in H_2_O/acetonitrile (50/50 vol % containing 0.05 M NaNO_3_). ^d^ Calculated from *M*_n_ of poly(NVI-Br) macro-CTA and comonomer composition determined by ^1^H NMR in DMSO-*d*_6_.

## Supplementary Materials

The following are available online at www.mdpi.com/2073-4360/9/11/616/s1. Figure S1: 1H NMR spectra of (a) *N*-methyl ethylimidzolium bromide (MEI-Br) and (b) MEI-NTf_2_ in DMSO-d_6_; Figure S2: 13C NMR spectra of (a) MEI-Br and (b) MEI-NTf_2_ in DMSO-*d*_6_; Figure S3: 1H NMR spectra of (a) poly(NVI-Br) and (b) poly(NVI-NTf_2_) in DMSO-*d*_6_; Figure S4: SEC traces of poly(NVI-Br)_68_-*b*-poly(NIPAM)_32_ (blue line) and poly(NVI-Br) macro-CTA (red line); Figure S5: 13C NMR spectra of (a) poly(NVI-Br)-*b*-poly(NIPAM) and (b) poly(NVI-NTf_2_)-*b*-poly(NIPAM) in DMSO-*d*6; Figure S6: TG curves of poly(NVI-Br)-*b*-poly(NIPAM) and poly(NVI-NTf_2_)-*b*-poly(NIPAM) under nitrogen atmosphere; Figure S7: Temperature-dependent ionic conductivity of poly(NVI-NTf2)_48_-*b*-poly(NIPAM)_52_. Acetone solution of the block copolymer was casted onto a platinum electrode and dried at 40 °C for 2 h. After it was allowed at room temperature overnight, the sample was dried at 90 °C for 2 h; Figure S8: DSC curves of (a) poly(NVI-NTf_2_) and (b) poly(NVI-NTf_2_)-*b*-poly(NIPAM); Figure S9: DLS profiles of poly(NVI-Br)_21_-*b*-poly(NIPAM)_79_ in aqueous solution (polymer conc. = 2.0 mg/mL) at 25 °C and 40 °C; Figure S10: SFM (a,b) height and (c,d) phase images of poly(NVI-Br)-*b*-poly(NIPAM)s; NVI-Br/NIPAM = (a,c) 68/32 and (b,d) 21/79, respectively. The samples were prepared by the drop casting of the methanol solutions of the block copolymers onto mica substrates; Table S1: Synthesis of poly(NVI-Br)-*b*-poly(NIPAM) by RAFT polymerization of NIPAM using the dithiocarbamate-terminated poly(NVI-Br) macro-CTA with AIBN in methanol ^a^; Table S2: Solubility of homopolymers and block copolymers.
