# Supplementary material for: Ionic Conductivity and Assembled Structures of Imidazolium Salt-Based Block Copolymers with Thermoresponsive Segments
Source: Polymers (Basel). 2017 Nov 15;9(11):616. doi: 10.3390/polym9110616 (PMC6418687; doi:10.3390/polym9110616)
Supplement: Supplementary file 1 [file polymers-09-00616-s001.pdf]

polymers

MDPI

Supplementary Materials: Ionic Conductivity and Assembled Structures of Imidazolium Salt-Based Block Copolymers with Thermoresponsive Segments

Kazuhiro Nakabayashi [1,2], Yu Sato [1], Yuta Isawa [2], Chen-Tsyr Lo [2] and Hideharu Mori *[,1,2]

[1] Department of Polymer Science and Engineering, Graduate School of Science and Engineering, Yamagata University, 4-3-16, Jonan, Yonezawa 992-8510, Japan; nakabayashi.k@yz.yamagata-u.ac.jp (K.N.); txa16692@st.yamagata-u.ac.jp (Y.S.);

[2] Department of Organic Materials Science, Graduate School of Organic Materials Science, Yamagata University, 4-3-16, Jonan, Yonezawa 992-8510, Japan; tkh21629@st.yamagata-u.ac.jp (Y.I.); ct.lo@yz.yamagata-u.ac.jp (C.-T.L.)

* Correspondence: h.mori@yz.yamagata-u.ac.jp; Tel.: +81-238-26-3765; Fax: +81-238-26-3092

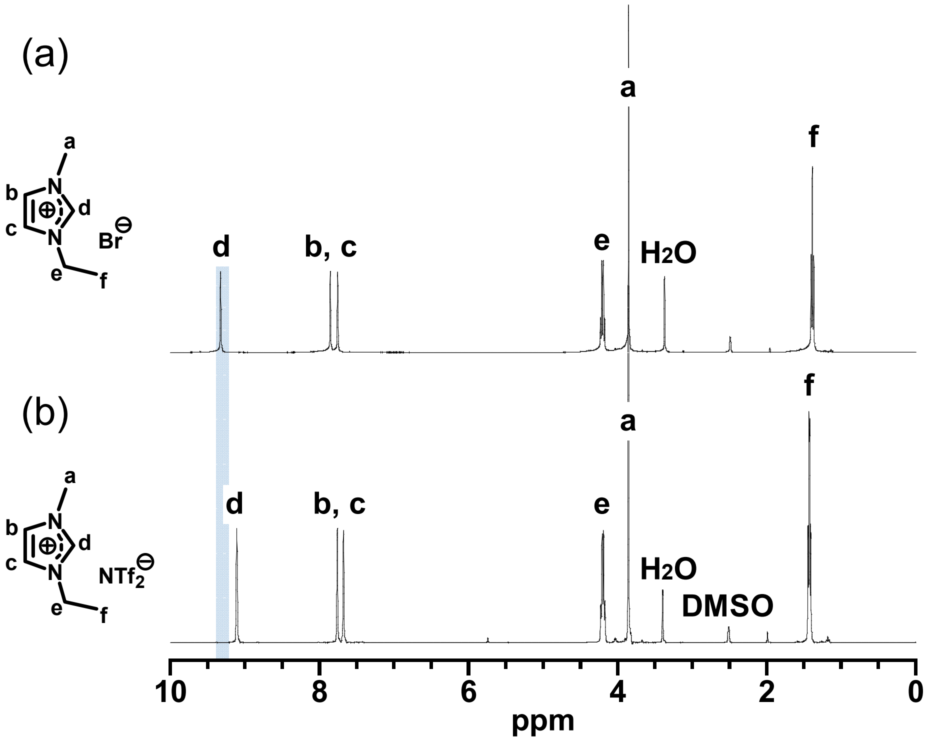

Figure S1. ^{1}H NMR spectra of (a) *N*-methyl ethylimidzolium bromide (MEI-Br) and (b) MEI-NTf₂ in DMSO-*d*₆.

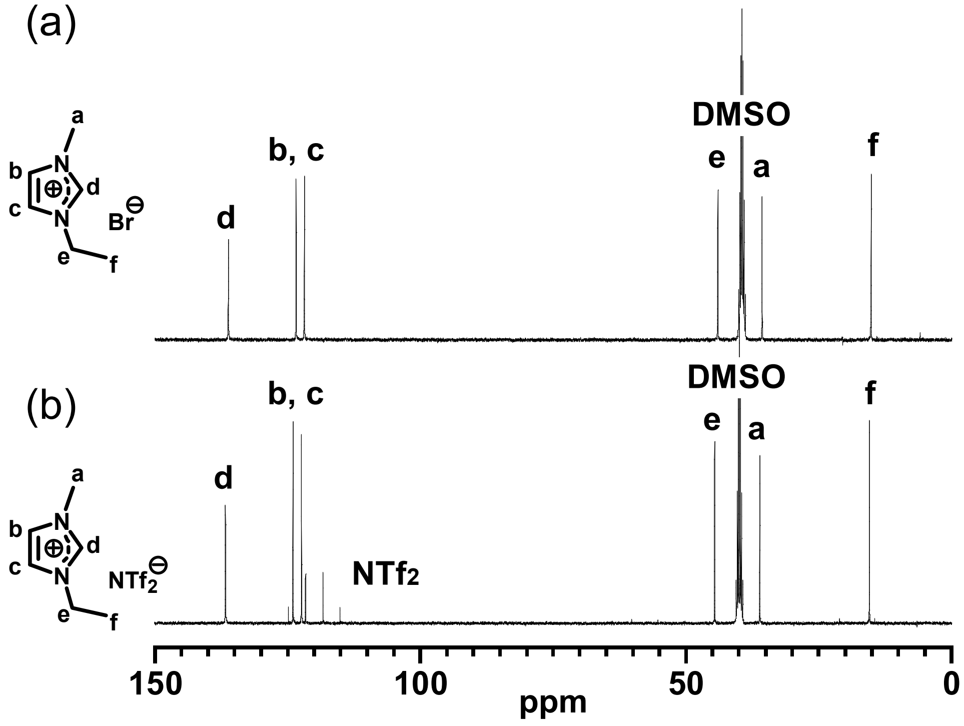

Figure S2. ^{13}C NMR spectra of (a) MEI-Br and (b) MEI-NTf_2 in DMSO-d_6.

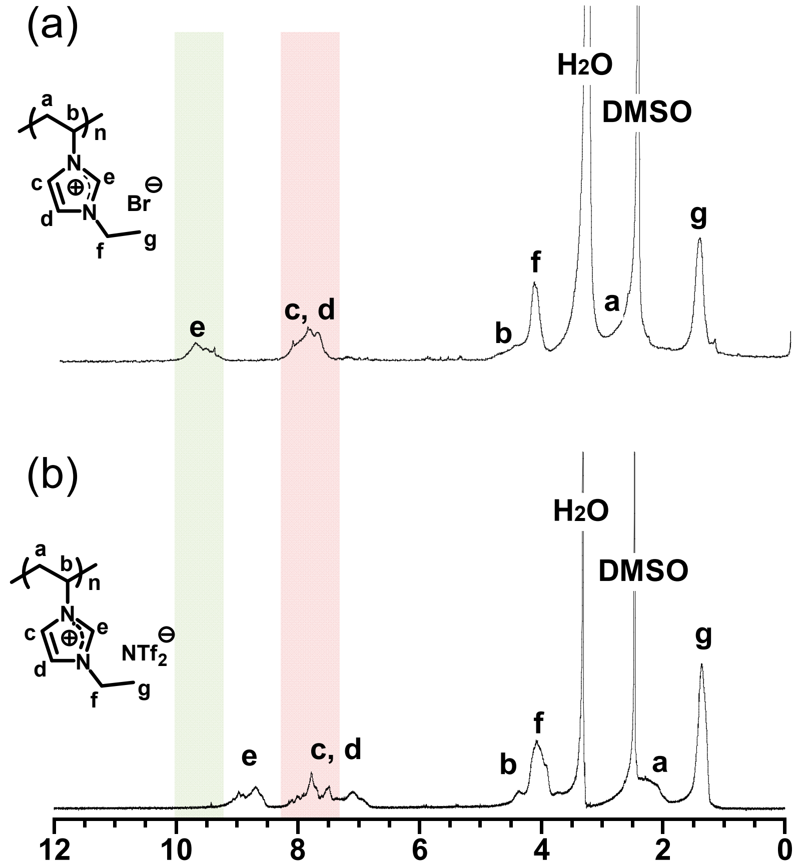

Figure S3. ^1H NMR spectra of (a) poly(NVI-Br) and (b) poly(NVI-NTf_2) in DMSO-d_6.

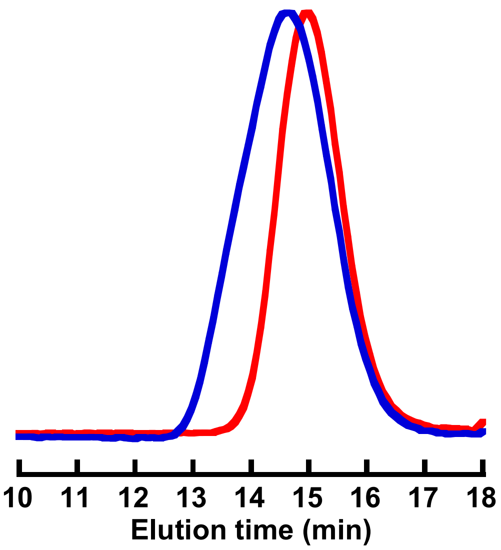

Figure S4. SEC traces of poly(NVI-Br)$_{68}$-*b*-poly(NIPAM)$_{32}$ (blue line) and poly(NVI-Br) macro-CTA (red line).

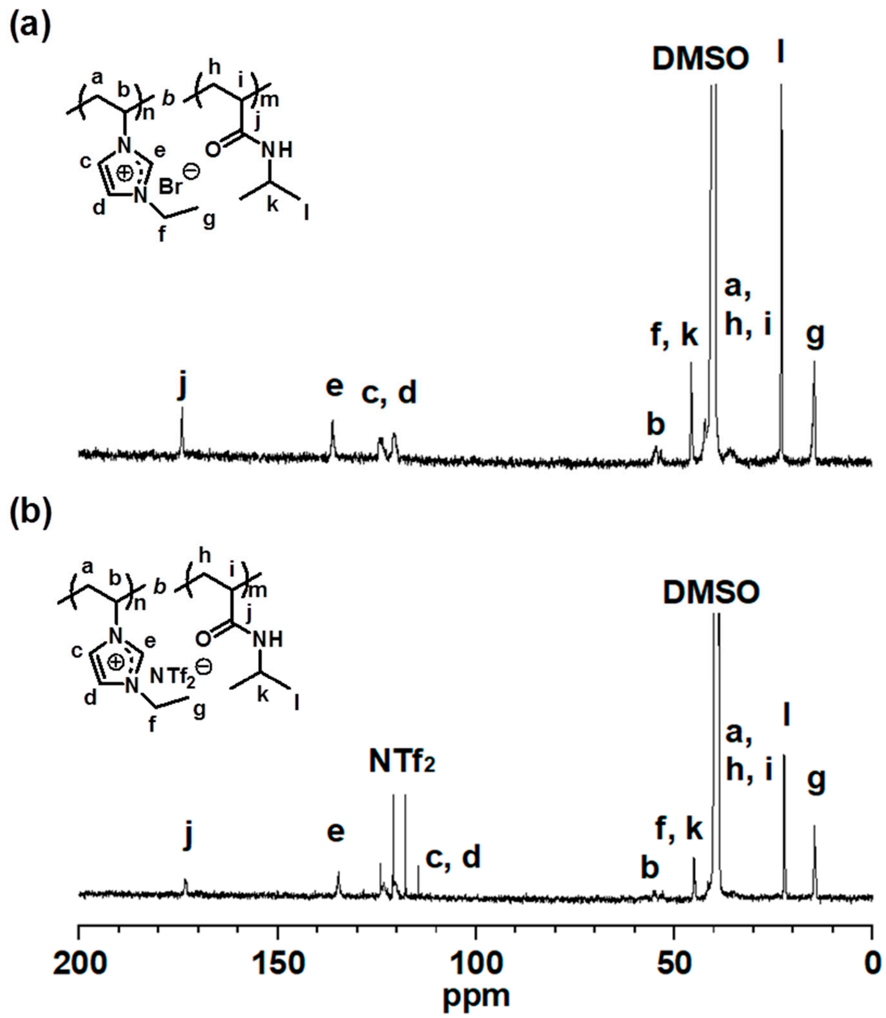

Figure S5. ^{13}C NMR spectra of (a) poly(NVI-Br)-*b*-poly(NIPAM) and (b) poly(NVI-NTf_2)-*b*-poly(NIPAM) in DMSO-d_6.

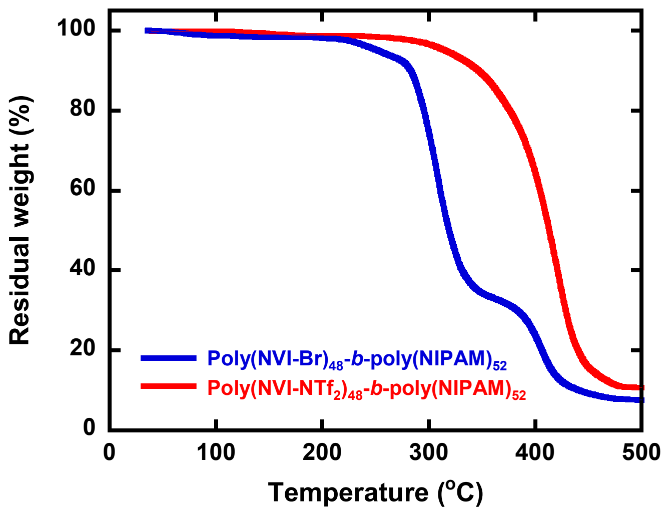

Figure S6. TG curves of poly(NVI-Br)-*b*-poly(NIPAM) and poly(NVI-NTf2)-*b*-poly(NIPAM) under nitrogen atmosphere.

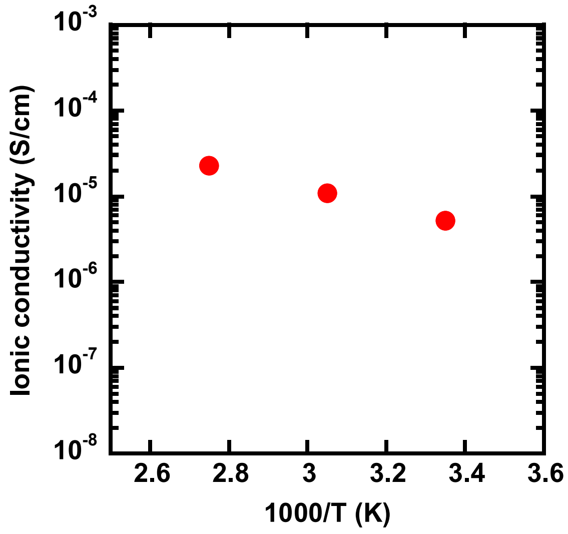

Figure S7. Temperature-dependent ionic conductivity of poly(NVI-NTf2)48-*b*-poly(NIPAM)52. Acetone solution of the block copolymer was casted onto a platinum electrode and dried at 40 °C for 2 h. After it was allowed at room temperature overnight, the sample was dried at 90 °C for 2 h.

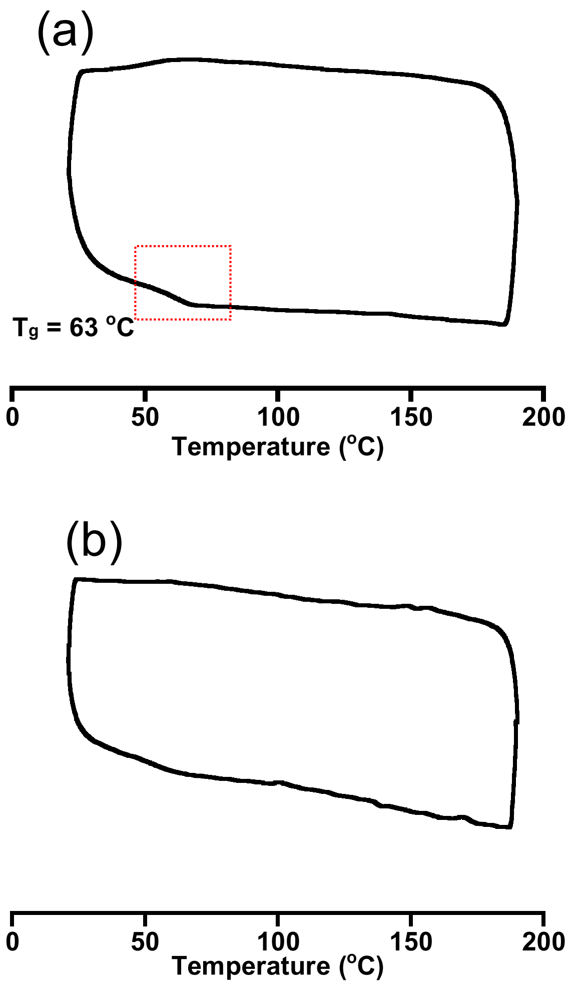

Figure S8. DSC curves of (a) poly(NVI-NTf_2) and (b) poly(NVI-NTf_2)-*b*-poly(NIPAM).

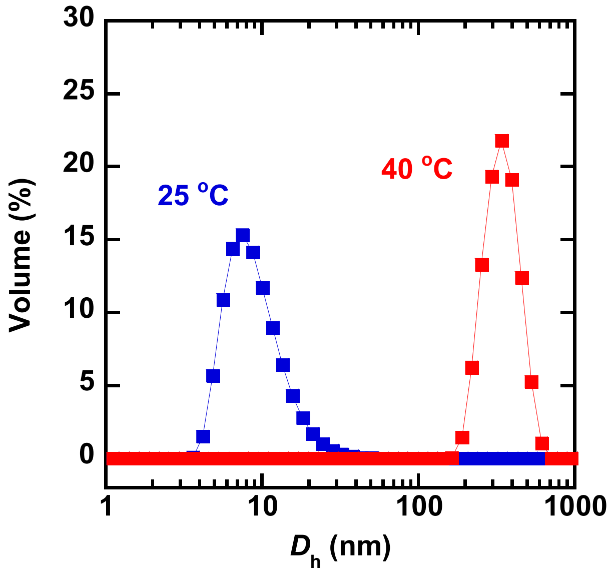

Figure S9. DLS profiles of poly(NVI-Br)$_{21}$-*b*-poly(NIPAM)$_{79}$ in aqueous solution (polymer conc. = 2.0 mg/mL) at 25 °C and 40 °C.

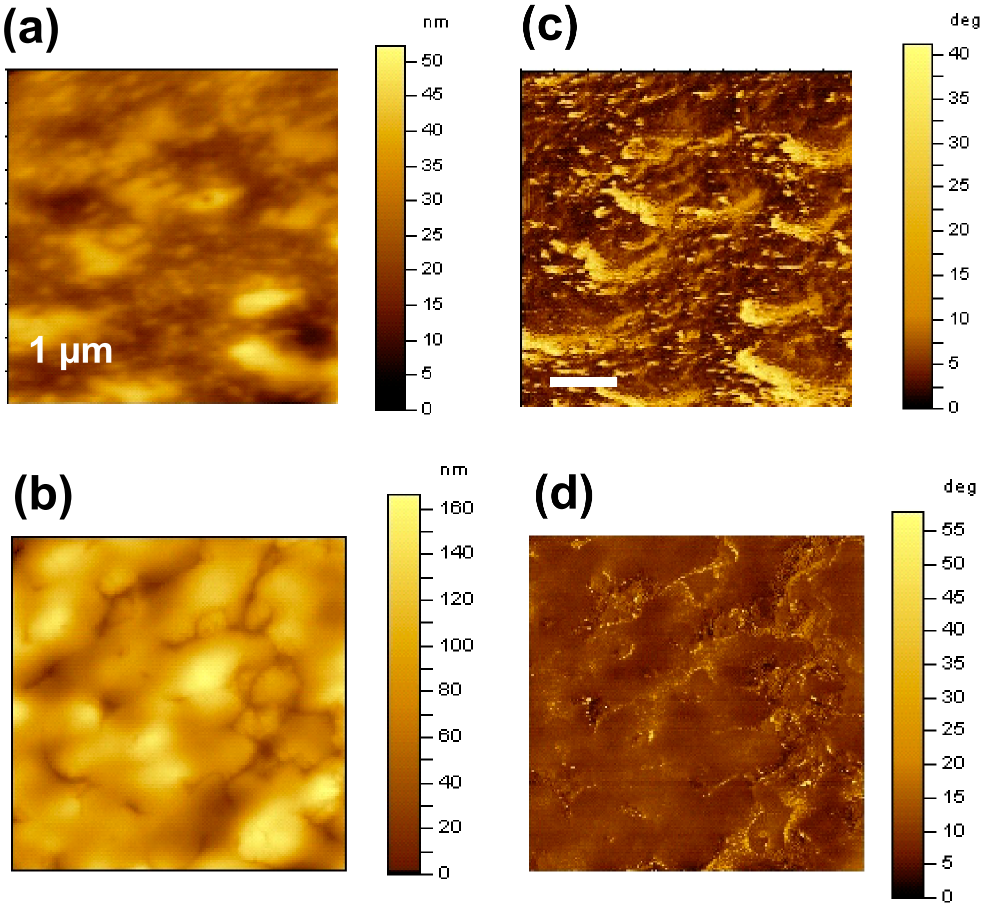

Figure S10. SFM (a-b) height and (c-d) phase images of poly(NVI-Br)-*b*-poly(NIPAM)s; NVI-Br/NIPAM = (a, c) 68/32 and (b, d) 21/79, respectively. The samples were prepared by the drop casting of the methanol solutions of the block copolymers onto mica substrates.

Table S1. Synthesis of poly(NVI-Br)-*b*-poly(NIPAM) by RAFT polymerization of NIPAM using the dithiocarbamate-terminated poly(NVI-Br) macro-CTA with AIBN in methanol [a].

Macro-CTA (M_n, M_w/M_n)	$[M]_0$/ $[\text{macro-CTA}]_0$	Conv.[d]/ Yield[e] (%)	M_n [f] (theory)	M_n [d] (NMR)	M_n [g] (SEC)	M_w/M_n [g] (SEC)	n : m [d]
15,400, 1.27	50 [b]	53/78	18,900	19,500	20.600	1.39	68 : 32
7,600, 1.11	50 [c]	94/75	13,700	13,000	9,100	1.26	48 : 52
14,000, 1.30	400 [c]	89/70	60,000	46,900	18,000	1.38	21 : 79

[a] Polymerization was carried out at 80 °C for 6h, $[M]_0$ = 0.1 g/mL. [b] $[\text{macro-CTA}]_0/[I]_0$ = 2. [c] $[\text{macro-CTA}]_0/[I]_0$ = 0.5. [d] Calculated by ^1H NMR in DMSO-d_6. [e] Diethyl ether-insoluble part. [f] The theoretical molecular weight ($M_{n,\text{theory}}$) = (MW of M) × $[M]_0/[\text{macro-CTA}]_0$ × conv. + (MW of macro-CTA). [g] Measured by SEC using poly (ethylene oxide) standards in H_2O/acetonitrile (50/50 vol% containing 0.05 M $NaNO_3$).

Table S2. Solubility of homopolymers and block copolymers.

	H_2O	DMF	DMSO	MeOH	Acetone	THF
Poly(NVI-Br)	+	+	+	+	-	-
Poly(NVI-NTf_2)	-	+	+	-	+	-
Poly(NIPAM)	+	+	+	+	+	+
Poly(NVI-Br)$_{48}$-*b*-poly(NIPAM)$_{52}$	+	-	+	+	-	-
Poly(NVI-NTf_2)$_{48}$-*b*-poly(NIPAM)$_{52}$	-	+	+	+	+	-
	Dioxane	$CHCl_3$	CH_2Cl_2	AcOEt	Hexane	Ether
Poly(NVI-Br)	-	-	-	-	-	-
Poly(NVI-NTf_2)	-	-	-	-	-	-
Poly(NIPAM)	+	+	+	+	-	-
Poly(NVI-Br)$_{48}$-*b*-poly(NIPAM)$_{52}$	-	-	-	-	-	-
Poly(NVI-NTf_2)$_{48}$-*b*-poly(NIPAM)$_{52}$	-	-	-	-	-	-

+ : Soluble at room temperature, - : Insoluble, ± : Partially soluble

© 2017 by the authors. Submitted for possible open access publication under the terms and conditions of the Creative Commons Attribution (CC BY) license (http://creativecommons.org/licenses/by/4.0/).
